# Conventional and phenomics characterization provides insight into the diversity and relationships of hypervariable scarlet (*Solanum aethiopicum* L.) and gboma (*S. macrocarpon* L.) eggplant complexes

**DOI:** 10.3389/fpls.2014.00318

**Published:** 2014-07-07

**Authors:** Mariola Plazas, Isabel Andújar, Santiago Vilanova, Pietro Gramazio, F. Javier Herraiz, Jaime Prohens

**Affiliations:** Instituto de Conservación y Mejora de la Agrodiversidad Valenciana, Universitat Politècnica de ValènciaValència, Spain

**Keywords:** crop complexes, cultivar groups, descriptors, phenomics, *Solanum aethiopicum*, *Solanum macrocarpon*, Tomato Analyzer

## Abstract

Scarlet (*Solanum aethiopicum*) and gboma (*S. macrocarpon*) eggplants are major vegetable crops in sub-Saharan Africa. Together with their respective wild ancestors (*S. anguivi* and *S. dasyphyllum*) and intermediate cultivated-wild forms they constitute the so-called scarlet and gboma eggplant complexes. We used conventional descriptors and the high-throughput phenomics tool Tomato Analyzer for characterizing 63 accessions of the scarlet eggplant complex, including the four *S. aethiopicum* cultivar groups (Aculeatum, Gilo, Kumba, and Shum), Intermediate *S. aethiopicum*-*S. anguivi* forms, and *S. anguivi*, and 12 cultivated and wild accessions of the gboma eggplant complex. A large diversity was found between both complexes, showing that they are very well differentiated from each other. Within the scarlet eggplant complex, many significant differences were also found among cultivar groups, but more differences were found for fruit traits evaluated with Tomato Analyzer than with conventional descriptors. In particular, Tomato Analyzer phenomics characterization was useful for distinguishing small fruited groups (Shum, Intermediate, and *S. anguivi*), as well as groups for which few or no significant differences were observed for plant traits. Multivariate principal components analysis (PCA) separated well all groups, except the Intermediate group which plotted between *S. anguivi* and small fruited *S. aethiopicum* accessions. For the gboma eggplant complex, *S. dasyphyllum* was clearly distinguished from *S. macrocarpon* and an important diversity was found in the latter. The results have shown that both complexes are hypervariable and have provided insight into their diversity and relationships. The information obtained has important implications for the conservation and management of genetic resources as well as for the selection and breeding of both scarlet and gboma eggplants.

## Introduction

The scarlet (*Solanum aethiopicum* L.) and gboma (*S. macrocarpon L.)* eggplants are two cultivated African vegetable crops locally important in its region of origin in tropical sub-Saharan Africa (Lester et al., 1990; Schippers, 2000; Lester and Daunay, 2003; Maundu et al., 2009). Scarlet eggplant is, together with tomato, onion, pepper and okra, one of the five most important vegetables in Central and West Africa (Schippers, 2000; Maundu et al., 2009). Gboma eggplant is in general less important than scarlet eggplant, although in some areas like in Benin and in the rain forest regions of Coastal Africa and Congo River, is one of the major vegetables (Lester et al., 1990; Dansi et al., 2008). Cultivation of both species is mostly restricted to Africa, but *S. aethiopicum* is also cultivated in the Caribbean and Brazil (Schippers, 2000), where it was probably brought by slaves, as well as in some areas of the south of Italy (Sunseri et al., 2010). Both scarlet and gboma eggplants are also important genetic resources for common eggplant (*S. melongena* L.) breeding, as the three species can be intercrossed giving hybrids with intermediate fertility (Daunay et al., 1991; Oyelana and Ugborogho, 2008; Prohens et al., 2012; Khan et al., 2013). Scarlet eggplant and its interspecific hybrids with *S. melongena* are also used as rootstocks for eggplant cultivation (Gisbert et al., 2011).

Within the genus *Solanum*, *S. aethiopicum* belongs to section Oliganthes (Lester, 1986; Lester and Niakan, 1986), while *S. macrocarpon* to section Melongena (Lester et al., 1990; Lester and Daunay, 2003; Lester et al., 2011). *Solanum aethiopicum* is a hypervariable species (i.e., characterized by many types and forms morphologically different), with hundreds of local varieties (Lester et al., 1986). Such morphological variability has resulted in about 20 different scientific names through the taxonomic history of this crop (Lester, 1986). Lester (1986) recognized four cultivar groups, namely Aculeatum, Gilo, Kumba, and Shum. The four cultivar groups of *S. aethiopicum* are completely interfertile (Lester and Niakan, 1986) and, although historically they have been treated as distinct species by several authors, it is generally accepted that they form part of a single species (Lester et al., 1986, 2011; Edmonds, 2012). Classification of accessions to their cultivar group can be made using a simple classification key (Lester et al., 1986). Regarding the utilization of each cultivar group, Aculeatum is used as an ornamental, Gilo for its fruits, Kumba for both fruits and leaves, and Shum for its leaves (Lester, 1986; Schippers, 2000; Lester and Daunay, 2003). The wild ancestor of *S. aethiopicum* is *S. anguivi* (Lester and Niakan, 1986). Hybrids between *S. aethiopicum* and *S. anguivi* Lam. are fully fertile (Lester and Niakan, 1986; Lester and Thitai, 1989). *Solanum aethiopicum* together with *S. anguivi* and their intermediate forms constitute the scarlet eggplant complex.

*Solanum macrocarpon* is also hypervariable in morphology, although to a lesser extent than *S. aethiopicum* (Lester and Daunay, 2003). Depending on the cultivar, *S. macrocarpon* is cultivated for its fruits, leaves or both (Schippers, 2000; Lester and Daunay, 2003; Maundu et al., 2009). *Solanum macrocarpon* was domesticated from the wild *S. dasyphyllum* Schum and Thonn. (Bukenya and Carasco, 1994). Both species are fully interfertile and together with their weedy intermediate forms for the gboma eggplant complex (Bukenya and Carasco, 1994).

Morphological characterization using conventional descriptors has proved useful for describing and establishing relationships among cultivar groups and accessions in scarlet and gboma eggplants (Lester et al., 1986; Polignano et al., 2010; Sunseri et al., 2010; Adeniji et al., 2012, 2013). These works have mostly focused on scarlet eggplant, revealing that it is a highly variable crop. The most comprehensive study was performed by Lester et al. (1986) who characterized 108 accessions of the scarlet eggplant complex using morphological and taxonomically relevant traits and found that the four cultivar groups could be distinguished by a syndrome of characteristics (i.e., a set of characteristics that are observed in a single group). These authors also found some accessions which were intermediate between *S. anguivi* and *S. aethiopicum* (Lester et al., 1986). The rest of characterization works (Polignano et al., 2010; Sunseri et al., 2010; Adeniji et al., 2012, 2013) involved fewer accessions and were based on morphological and agronomic descriptors. These latter studies found some degree of differentiation among the four *S. aethiopicum* groups, but considerable overlapping among groups was found. Many fewer studies have been devoted to the diversity of gboma eggplant. Polignano et al. (2010) evaluated 16 accessions of *S. macrocarpon* and found that it was a variable crop, which presented a continuous variation for the morphological diversity.

Modern phenomics tools may also be useful for precise characterization and for studying the diversity and relationships in collections of genetic resources (Furbank and Tester, 2011). In this respect, the high-throughput phenomics software tool Tomato Analyzer, which was initially developed for fruit shape analysis in tomato (Brewer et al., 2006, 2007; Gonzalo and van der Knaap, 2008; Rodríguez et al., 2010), has also proved useful for the detailed and accurate characterization of eggplant accessions (Hurtado et al., 2013) as well as for segregating generations between *S. melongena* and *S. aethiopicum* (Prohens et al., 2012). Tomato Analyzer allows scoring a large number of fruit shape traits from scanned images of fruit sections and is a powerful tool for precise description of fruit morphology. Therefore, Tomato Analyzer may be useful for the fruit shape characterization of germplasm collections of scarlet and gboma eggplants. Furthermore, fruit shape is considered as a very important trait in the preferences of farmers in selecting a variety of scarlet or gboma eggplant (Adeniji and Aloyce, 2012) and in consequence is a trait of major importance in these two crops, especially in varieties used for their fruits.

In this work, we characterize a collection of accessions of the scarlet and gboma eggplants complexes using conventional and phenomics (Tomato Analyzer) descriptors. The objective is to provide phenotypic information of relevance on the diversity and relationships of the two crops and their cultivar groups. This information will be useful for the classification, management of genetic resources, selection and breeding of both crops.

## Materials and methods

### Plant material

**Figure 1:**
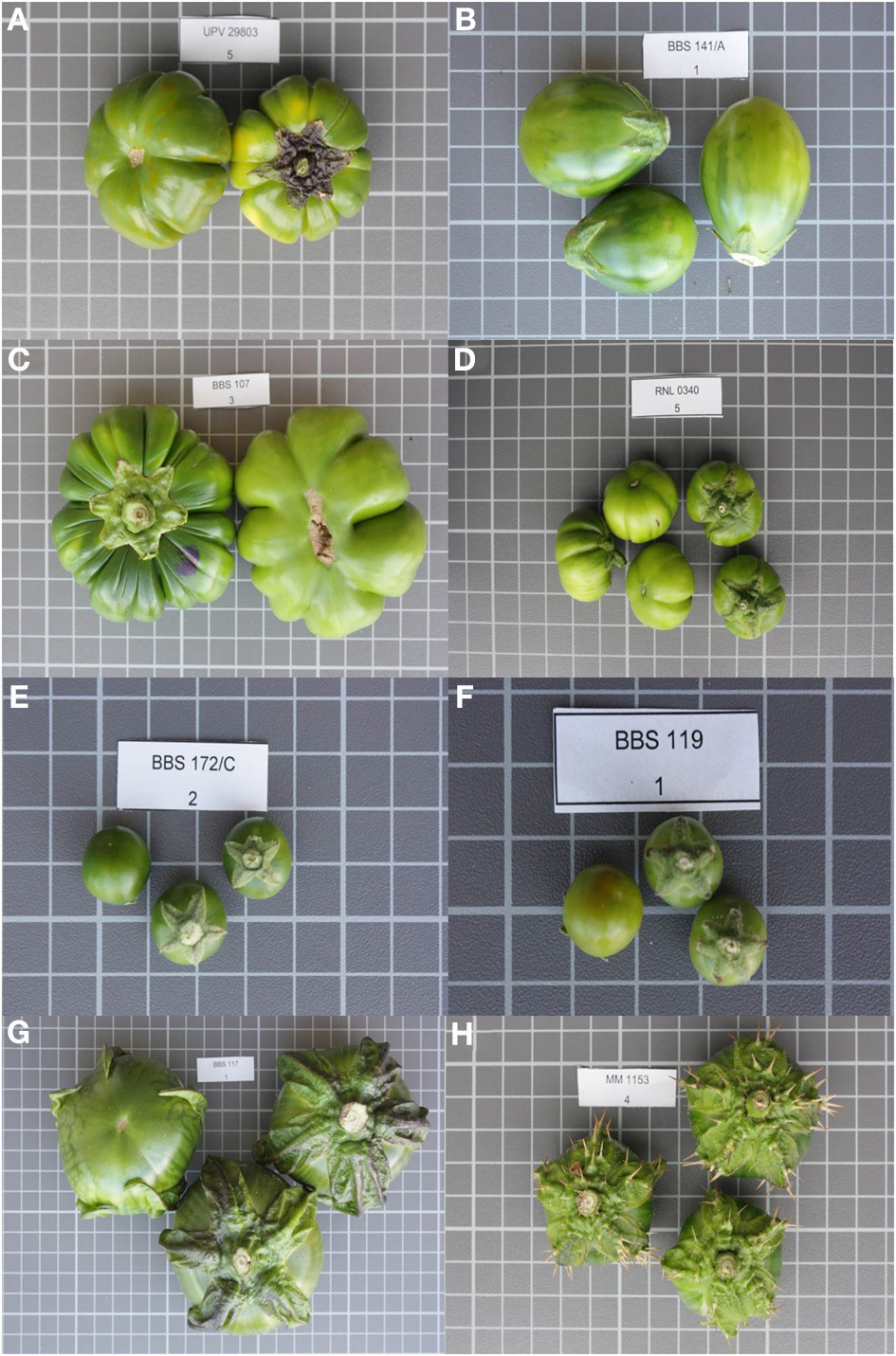
**Representative fruits of each of the scarlet eggplant complex (*S. aethiopicum* and *S. anguivi*) and gboma eggplant complex (*S. macrocarpon* and *S. dasyphyllum*) groups evaluated**. Groups include *S. aethiopicum* groups Aculeatum **(A)**, Gilo **(B)**, Kumba **(C)**, Shum **(D)**, Intermediate beween *S. aethiopicum* and *S. anguivi* **(E)**, *S. anguivi* **(F)**, *S. macrocarpon* **(G)**, and *S. dasyphyllum* **(H)**. Fruits are not depicted at the same scale; the size of the grid cells is 1 × 1 cm.

Sixty-three accessions of the scarlet eggplant complex (*S. aethiopicum* and *S. anguivi*) and 12 accessions of the gboma eggplant complex (*S. macrocarpon* and *S. dasyphyllum*) from the germplasm bank of the Universitat Politècnica de València (València, Spain) were used for the present study. The scarlet eggplant complex accessions were classified according to the key to taxa of *S. aethiopicum* and *S. anguivi* established by Lester et al. (1986), which includes *S. aethiopicum* groups Aculeatum, Gilo, Kumba, and Shum, and *S. anguivi* (Figure 1). Accessions that could not be allocated to any of the groups, as they shared intermediate characteristics between *S. aethiopicum* and *S. anguivi* were assigned to a group denominated Intermediate. Gboma eggplant complex accessions were classified as *S. macrocarpon* or *S. dasyphyllum* according to the key of Lester et al. (2011) (Figure 1).

### Characterization

Individual plants were characterized using 18 plant descriptors commonly used for cultivated eggplant species and wild relatives characterization (IBPGR, 1990; Prohens et al., 2005; van der Weerden and Barendse, 2007; Polignano et al., 2010; Prohens et al., 2012). Plant descriptors include traits related to whole plant (5), leaves (7), and inflorescences and flowers (6) (Table 1). Seven plant descriptors are metric, four are meristic (traits in which the number of parts or components are counted), and seven are measured in a quantitative scale. For each individual plant, several commercially ripe (i.e., physiologically immature, see Figure 1) fruits were weighted, manually measured for length and breadth, and longitudinally cut and scanned with an HP Scanjet G4010 photo scanner (Hewlett-Packard, Palo Alto, CA, USA) at a resolution of 300 dpi and subjected to morphometric analysis with Tomato Analyzer version 3 software (Rodríguez et al., 2010). Data were recorded for a total of 27 fruit descriptors (Table 1), of which three were manually measured (Weight, Length, Breadth) and 24 were automatically obtained with Tomato Analyzer, including basic (6), fruit shape index (2), blockiness (3), homogeneity (3), proximal fruit end shape (1), distal fruit end shape (1), asymmetry (3), and internal eccentricity (5) descriptors. Nine fruit traits had units and 18 were unitless. All fruit descriptors were metric. Default settings were used for blockiness and proximal fruit end shape and distal fruit end shape descriptors (Rodríguez et al., 2010). A complete description of these traits can be found elsewhere (Rodríguez et al., 2010; Prohens et al., 2012; Hurtado et al., 2013).

**Table 1:** **Plant and fruit descriptors used for the characterization of a collection of scarlet eggplant complex (*S. aethiopicum* and *S. anguivi*) and gboma eggplant complex (*S. macrocarpon* and *S. dasyphyllum*) accessions**.

**Descriptors**	**Units/scale/description**
**PLANT DESCRIPTORS**	
Plant Height	cm
Hypocotyl Anthocyanins Intensity	(S) 0 = Absent; 9 = Very strong
Shoot Tip Anthocyanins Intensity	(S) 0 = Absent; 9 = Very strong
Stem Diameter	cm
Angle Between Main Branches	(S) 1 ≤ 40°; 5 > 50°
Leaf Pedicel Length	cm
Leaf Blade Length	cm
Leaf Blade Breadth	cm
Leaf Blade Lobing	(S) 1 = Very weak; 9 = Very strong
Leaf Surface Shape	(S) 1 = Flat; 9 = Very convex or bullate
Leaf Prickles	(S) 0 = None; 9 = Very many (>20)
Length of Largest Leaf Prickle	cm
Number of Flowers per Inflorescence	(M)—
Corolla Color	(S)1 = Greenish white; 9 = Bluish violet
Number of Sepals	(M)—
Number of Petals	(M)—
Number of Stamens	(M)—
Corolla Diameter	mm
**FRUIT DESCRIPTORS**	
Weight	g
Length	cm
Breadth	cm
Perimeter	cm
Area	cm^2^
Width Mid-height	The width measured at ½ of the fruit's height (cm)
Maximum Width	The maximum horizontal distance of the fruit (cm)
Height Mid-width	The height measured at ½ of the fruit's width (cm)
Maximum Height	The maximum vertical distance of the fruit (cm)
Fruit Shape Index External I	The ratio of the Maximum Height to Maximum Width
Fruit Shape Index External II	The ratio of the Height Mid-width to Width Mid-height
Proximal Fruit Blockiness	Ratio of the width at the upper blockiness position to Width_MH
Distal Fruit Blockiness	Ratio of the width at the lower blockiness position to Width_MH
Fruit Shape Triangle	Ratio of the width at the upper blockiness position to the lower blockiness position
Ellipsoid	The ratio of the error resulting from a best-fit ellipse to the area of the fruit; smaller values indicate that the fruit is more ellipsoid
Circular	The ratio of the error resulting from a best-fit circle to the area of the fruit; smaller values indicate that the fruit is more circular
Rectangular	The ratio of the rectangle bounding the fruit to the rectangle bounded by the fruit
Shoulder Height	The ratio of the average height of the shoulder points above the proximal end point to Maximum Height
Distal End Protrusion	Ratio of the area of the distal protrusion to the total area of the fruit, multiplied by 10
Obovoid	Calculated according to the formula provided in the tomato Analyzer Manual (Rodríguez et al., 2010). The higher the value, the greater is the area of the fruit below mid height
Ovoid	Calculated according to the formula provided in the tomato Analyzer Manual (Rodríguez et al., 2010). The higher the value, the greater is the area of the fruit above mid height
Width Widest Pos	The ratio of the height at which the Max_Width occurs to the Max_Height
Eccentricity	The ratio of the height of the internal ellipse to the Maximum Height
Proximal Eccentricity	The ratio of the area of the height of the internal ellipse to the distance between the bottom of the ellipse and the top of the fruit
Distal Eccentricity	The ratio of the area of the height of the internal ellipse to the distance between the bottom of the ellipse and the bottom of the fruit
Fruit Shape Index Internal	The ratio of the internal ellipse's height to its width
Eccentricity Area Index	The ratio of the area of the fruit outside the ellipse to the total area of the fruit

*Full details of the descriptors can be consulted elsewhere (IBPGR, 1990; Prohens et al., 2005; Brewer et al., 2006, 2007; van der Weerden and Barendse, 2007; Darrigues et al., 2008; Gonzalo and van der Knaap, 2008; Rodríguez et al., 2010). Thirty-four descriptors are metric, four are meristic, and seven are measured in a quantitative scale. The two latter are indicated by an “M” and a “Q,” respectively in their description*.

### Data analyses

The mean, range, and coefficient of variation (CV) values for plant and fruit descriptors were calculated for each of the scarlet eggplant and gboma eggplant complexes. Two-tailed *t* tests were performed on mean values for each descriptor in order to study signification of differences between means of scarlet and gboma eggplant complexes (Little and Hills, 1978). Analyses of variance (ANOVA) tests for each of the scarlet eggplant and gboma eggplant complexes were performed on plant and fruit values to detect differences among groups within each complex. For descriptors in which the mean was proportional to standard deviation, log transformed data were used for the ANOVA tests in order to avoid scaling effects (Little and Hills, 1978). Significant (*P* < 0.05) differences among group means were detected using the Student-Newman-Keuls multiple range test. No corrections were performed for controlling type I error (false positives) derived from multiple testing (Snedecor and Cochran, 1989). The number of significant differences between pairs of both scarlet eggplant and gboma eggplant groups means for plant and fruit descriptors were calculated. Principal components analysis (PCA) were performed using pairwise Euclidean distances among accession means.

## Results

### Diversity and differences between scarlet and gboma eggplant complexes

**Figure 2:**
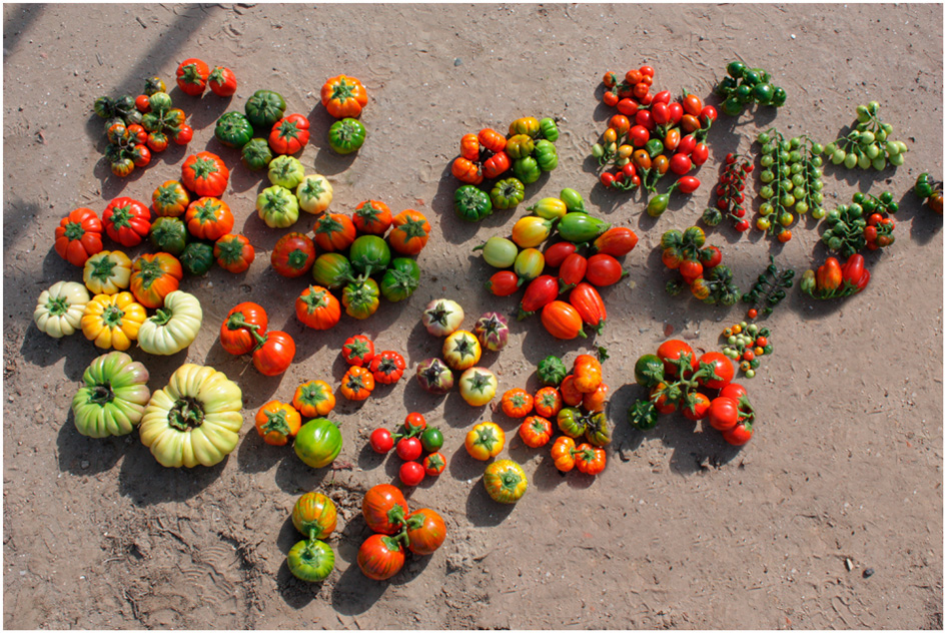
**Diversity among accessions of scarlet eggplant complex (*S. aethiopicum* and *S. anguivi*) in the evaluated collection**. Red fruits are physiologically mature.

**Figure 3:**
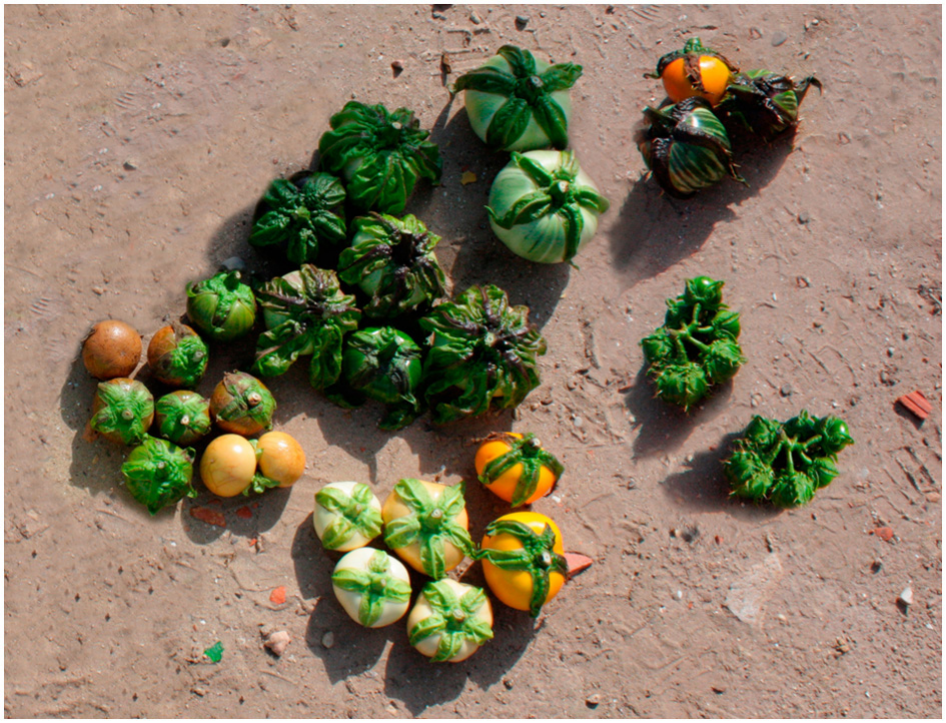
**Diversity among accessions of gboma eggplant complex (*S. macrocarpon* and *S. dasyphyllum*) eggplant in the evaluated collection**. Yellow and brown fruits are physiologically mature.

The morphological characterization of scarlet and gboma eggplant complexes revealed that the collection studied was phenotypically very diverse (Figures 2, 3). Both scarlet and gboma eggplant complexes displayed considerable diversity for most plant and fruit descriptors (Table 2). For plant traits measured in a scale, in both complexes the range of variation covers all or most of the scale range, with the exception of Corolla Color, which in the scarlet eggplant complex presents low values of the scale, while in the gboma eggplant complex it presents intermediate-high values (Table 2). For the rest of plant traits, the range of variation was broad in both complexes. For both complexes, the largest values of CV were found for the two anthocyanin intensity and the two prickliness traits, with values always above 100% and up to 328.6% for Length of Largest Prickle in the scarlet eggplant complex (Table 2). Also, in both complexes the traits with lowest CV values were the Number of Sepals, Number of Petals, and Number of Stamens, with CV values always below 15%. For 12 out of the 18 plant traits, the CV value was higher in the scarlet eggplant complex than in the gboma eggplant complex (Table 2).

**Table 2:** **Mean, range, and coefficient of variation (CV; %) for the plant and fruit descriptors studied in the scarlet eggplant complex (*S. aethiopicum* and *S. anguivi*) and gboma eggplant complex (*S. macrocarpon* and *S. dasyphyllum*) accessions, and significance of the differences between complex means**.

**Descriptors^a^**	**Scarlet eggplant (*n* = 63)**			**Gboma eggplant (*n* = 12)**		
	**Mean**	**Range**	**CV**	**Mean^b^**	**Range**	**CV**
**PLANT DESCRIPTORS**						
Plant Height (cm)	148	74–208	20.3	95^***^	50-129	26.3
Hypocotyl Anthocyanins Intensity	1.44	0.00–9.00	195.8	1.00^ns^	0.00–7.00	209.0
Shoot Tip Anthocyanins Intensity	1.52	0.00–9.00	180.3	1.17^ns^	0.00–7.00	199.1
Stem Diameter (cm)	2.77	1.50–4.20	23.1	2.66^ns^	2.00–3.25	15.0
Angle Between Main Branches	2.65	1.00–5.00	35.8	3.84^***^	1.00–5.00	34.9
Leaf Pedicel Length (cm)	5.66	2.33–12.80	40.3	1.59^***^	0.53–2.67	56.0
Leaf Blade Length (mm)	21.9	9.67–34.2	20.0	33.3^***^	26.2–40.0	14.2
Leaf Blade Breadth (cm)	16.7	7.0–31.0	24.6	20.0^*^	14.8–25.0	19.5
Leaf Blade Lobing	4.40	1.00–7.40	25.0	6.13^***^	4.20–9.00	26.3
Leaf Surface Shape	2.59	1.00–5.80	74.9	5.00^***^	1.00–9.00	34.2
Leaf Prickles	0.48	0.00–6.60	322.9	1.66^*^	0.00–9.00	163.3
Length of Largest Leaf Prickle (cm)	0.14	0.00–2.06	328.6	0.42^ns^	0.00–1.45	131.0
Number of Flowers per Inflorescence	3.34	1.00–12.6	83.5	3.66^ns^	1.20–5.40	36.9
Corolla Color	2.81	1.00–3.50	25.6	6.00^***^	5.00–7.00	17.3
Number of Sepals	5.66	5.00–7.00	9.5	5.27^*^	5.00–6.00	7.2
Number of Petals	5.62	5.00–8.00	10.7	5.19^*^	5.00–6.00	7.3
Number of Stamens	5.98	5.00–8.00	14.9	5.29^*^	5.00–6.00	8.1
Corolla Diameter (mm)	19.2	11.1–33.3	21.4	38.0^***^	23.5–55.3	23.9
**FRUIT DESCRIPTORS**						
Weight (g)	48	1–351	147.9	111^**^	22–177	43.2
Length (cm)	3.59	1.10–7.65	36.2	4.99^***^	2.97–6.70	19.6
Breadth (cm)	4.42	1.16–11.14	50.0	6.66^**^	3.83–8.46	20.6
Perimeter (cm)	13.9	3.9–30.0	41.0	20.2^***^	11.9–25.2	20.8
Area (cm^2^)	13.0	1.1–40.3	66.9	27.1^***^	9.3–41.4	37.6
Width Mid-height (cm)	4.20	1.21–9.72	45.7	6.55^***^	3.79–8.44	23.1
Maximum Width (cm)	4.24	1.21–9.90	46.2	6.60^***^	3.81–8.49	23.0
Height Mid-width (cm)	3.18	1.07–7.06	39.6	4.58^***^	2.94–6.79	23.6
Maximum Height (cm)	3.58	1.10–7.17	36.0	4.96^***^	3.03–6.92	23.0
Fruit Shape Index External I	0.93	0.54–1.89	36.6	0.77^ns^	0.68–1.34	23.4
Fruit Shape Index External II	0.87	0.32–1.92	46.0	0.72^ns^	0.60–1.32	26.4
Proximal Fruit Blockiness	0.66	0.46–0.79	12.1	0.74^***^	0.68–0.80	5.4
Distal Fruit Blockiness	0.64	0.39–0.76	12.5	0.62^ns^	0.57–0.65	3.2
Fruit Shape Triangle	1.07	0.69–1.76	18.7	1.20^*^	1.07–1.32	6.7
Ellipsoid	0.07	0.02–0.33	100.0	0.05^ns^	0.03–0.07	20.0
Circular	0.14	0.02–0.38	64.3	0.15^ns^	0.08–0.28	33.3
Rectangular	0.52	0.44–0.61	7.7	0.52^ns^	0.46–0.55	5.8
Shoulder Height	0.03	0.00–0.14	133.3	0.03^ns^	0.01–0.05	33.3
Distal End Protrusion	0.02	0.00–0.17	150.0	0.01^ns^	0.00–0.02	100.0
Obovoid	0.07	0.00–0.21	71.4	0.01^***^	0.00–0.05	100.0
Ovoid	0.08	0.00–0.23	75.0	0.15^***^	0.08–0.19	20.0
Width Widest Pos	0.49	0.39–0.59	8.2	0.45^**^	0.42–0.49	4.4
Eccentricity	0.71	0.44–0.79	14.1	0.74^ns^	0.69–0.78	4.1
Proximal Eccentricity	0.89	0.81–0.96	1.2	0.89^ns^	0.89–0.89	0.0
Distal Eccentricity	0.88	0.77–0.90	2.3	0.89^ns^	0.88–0.89	0.0
Fruit Shape Index Internal	0.86	0.31–1.60	44.2	0.72^ns^	0.60–1.32	26.4
Eccentricity Area Index	0.44	0.34–0.61	15.9	0.43^ns^	0.38–0.46	4.7

a *See Table 1 for definition of descriptors*.

b***, **, *, ns *indicate, respectively, significant at P < 0.001, P < 0.01, P < 0.05, or non-significant differences between scarlet and gboma eggplant complexes means, according to a two-tailed t-test*.

As occurred for the plant descriptors, a wide diversity was found for most fruit traits within each of the scarlet and gboma eggplant complexes (Table 2). In particular, for the nine fruit size traits evaluated (Weight to Maximum Height) the ranges of variation were very large. For example Weight, ranged between 1 and 351 g in the scarlet eggplant complex and between 22 and 177 g in the gboma eggplant complex. For the unitless fruit shape traits in most cases an important variation was found in both complexes, although in some cases (e.g., Proximal Eccentricity and Distal Eccentricity in the gboma eggplant complex) the range of variation was very limited (Table 2). CV values of 100% or larger were found for Weight, Ellipsoid, Shoulder Height, and Distal End Protrusion in the scarlet group and for Distal End Protrusion and Obovoid for the gboma eggplant complex. In both complexes, the lowest CV values were found for Proximal Eccentricity and Distal Eccentricity, with values for both traits of 0.0% in the gboma eggplant complex and as low as 1.2 and 2.3%, respectively, in the scarlet eggplant complex. For all fruit traits, with the exception of Obovoid, the CV was larger for the scarlet eggplant complex than for the gboma eggplant complex (Table 2).

Despite the wide diversity found within each of the scarlet eggplant and gboma eggplant complexes, many morphological significant (*P* < 0.05) differences existed for mean values between both complexes (Table 2). In this respect, when considering plant traits, on average, scarlet eggplants had plants that were taller (Plant Height), less erect (Angle Between Main Branches), with smaller leaf blade (Leaf Blade Length and Leaf Blade Breadth), less lobed leaves (Leaf Blade Loging), flatter leaf surface (Leaf Surface Shape), less prickly leaves (Leaf Prickles), greater number of flower parts (Number of Sepals, Number of Petals, and Number of Stamens), smaller flowers (Corolla Diameter), and longer leaf pedicel (Leaf Pedicel Length) than gboma eggplants (Table 2). Regarding fruit traits, the scarlet eggplant complex fruits were, on average, smaller (lower values for the nine fruit size traits), less blocky in the proximal part (Proximal Fruit Blockiness), less triangular (Fruit Shape Triangle), more obovoid (and less ovoid), and with highest values for the ratio of the height at which the maximum width occurs (Width Widest Pos) than the gboma eggplant complex fruits (Table 2).

### Differences and relationships among scarlet eggplant groups

Significant (*P* < 0.05) differences were found among the six scarlet eggplant complex groups for 15 out of the 18 plant traits evaluated (Table 3). The only exceptions were Stem Diameter, Leaf Blade Lobing, and Corolla Color. The number of significant differences between means of the scarlet eggplant complex groups for the 18 plant morphological traits evaluated range from 0 (between Gilo and Shum on one side and Intermediate and *S. anguivi* on the other) and 8 (between Aculeatum and Shum) (Table 4). Few differences in plant traits were also found between group Gilo on one side and groups Kumba, Intermediate and *S. anguivi* on the other, as well as between groups Shum, Intermediate and *S. anguivi* (Table 4). Among the most relevant differences found among scarlet eggplant complex groups for plant traits average values, plants of group Gilo were taller than those of group Kumba, group Aculeatum had higher anthocyanin content and prickliness than the other groups, groups Aculeatum, Intermediate and *S. anguivi* had more flowers per inflorescence than groups Gilo, Kumba, and Shum, group Kumba had larger flowers and higher number of flower parts than groups Shum, Intermediate and *S. anguivi*, and larger leaves than groups Shum and *S. anguivi* (Table 3).

**Table 3:** **Mean values for scarlet eggplant complex groups (*S. aethiopicum* groups Aculeatum, Gilo, Kumba, and Shum, Intermediate between *S. aethiopicum* and *S. anguivi*, and *S. anguivi*) for the plant descriptors for which significant (*P* < 0.05) differences have been found among group means**.

**Descriptors**	***S. aethiopicum***				**Intermediate**	***S. anguivi***	**Prob. F**
	**Aculeatum**	**Gilo**	**Kumba**	**Shum**			
*n*	5	37	10	2	8	1	
Plant Height (cm)	151^ab^	156^b^	114^a^	135^ab^	153^ab^	150^ab^	0.0033
Hypocotyl Anthocyanins Intensity	7.80^b^	0.31^a^	2.74^a^	0.50^a^	1.49^a^	0.00^a^	<0.0001
Shoot Tip Anthocyanins Intensity	7.80^b^	0.23^a^	2.98^a^	2.50^a^	1.68^a^	0.00^a^	<0.0001
Angle Between Main Branches	1.72^ab^	2.77^ab^	3.21^b^	2.00^ab^	2.35^ab^	1.00^a^	0.0134
Leaf Pedicel Length (cm)	7.88^b^	5.23^ab^	6.74^ab^	3.47^a^	5.82^ab^	3.00^a^	0.0342
Leaf Blade Length (mm)	23.8^bc^	21.5^abc^	25.5^c^	14.3^a^	20.9^abc^	15.5^ab^	0.0031
Leaf Blade Breadth (cm)	17.8^ab^	16.4^ab^	19.5^b^	10.5^a^	16.1^ab^	12.0^ab^	0.0412
Leaf Surface Shape	1.00^a^	2.54^ab^	3.88^ab^	4.33^b^	2.00^ab^	1.00^a^	0.0469
Leaf Prickles	5.32^b^	0.00^a^	0.00^a^	0.00^a^	0.47^a^	0.00^a^	<0.0001
Length of Largest Leaf Prickle (cm)	1.62^b^	0.00^a^	0.00^a^	0.00^a^	0.07^a^	0.00^a^	<0.0001
Number of Flowers per Inflorescence	7.18^b^	2.18^a^	1.97^a^	3.33^a^	7.51^b^	7.50^b^	<0.0001
Number of Sepals	5.97^ab^	5.60^ab^	6.33^b^	5.27^a^	5.06^a^	5.00^a^	<0.0001
Number of Petals	5.92^ab^	5.50^ab^	6.39^b^	5.33^ab^	5.16^a^	5.00^a^	<0.0001
Number of Stamens	6.01^ab^	5.91^ab^	7.18^b^	5.31^a^	5.06^a^	5.00^a^	<0.0001
Corolla Diameter (mm)	19.1^ab^	19.3^ab^	22.7^b^	12.5**^a^**	16.8^ab^	13.2^a^	0.0017

a *Means within rows separated by different letters are significantly different at P < 0.05, according to the Student-Newman-Keuls test*.

**Table 4:** **Number of significant (*P* < 0.05) differences among means for scarlet eggplant complex groups (*S. aethiopicum* groups Aculeatum, Gilo, Kumba, and Shum, Intermediate between *S. aethiopicum* and *S. anguivi*, and *S. anguivi*) for 18 conventional descriptors (above the diagonal) and for 27 Tomato Analyzer descriptors (below the diagonal)**.

	***S. aethiopicum***				**Intermediate**	***S. anguivi***
	**Aculeatum**	**Gilo**	**Kumba**	**Shum**		
***S. AETHIOPICUM***						
Aculeatum		5	5	8	4	5
Gilo	8		1	0	1	1
Kumba	7	13		5	4	7
Shum	12	9	14		1	2
Intermediate	15	9	16	0		0
*S. anguivi*	14	14	20	3	6	

For fruit traits, significant (*P* < 0.05) differences were found among the six scarlet eggplant complex groups for 24 out of the 27 fruit traits evaluated (Table 5). The number of significant differences among groups for fruit traits ranged from zero (between groups Shum and Intermediate) to 20 (between groups Kumba and *S. anguivi*) (Table 4). As occurred for plant traits, groups Shum, Intermediate and *S. anguivi* presented few differences (between 0 and 6). The rest of pairwise comparisons between groups presented at least 7 differences (Table 4). For the nine fruit size traits, in general the Kumba group presented the largest values, followed by groups Aculeatum and Gilo, then the groups Shum and Intermediate, and finally by *S. anguivi*, which presented the smallest fruits (Table 5). When considering fruit shape traits, the most relevant differences were that groups Aculeatum and Kumba had fruits more flattened (Fruit shape Index External I and II) than groups Gilo and Intermediate, group Aculeatum presented higher values for Proximal Fruit Blockiness than *S. anguivi* and of Distal Fruit Blockiness than groups Kumba and Intermediate, group Kumba was characterized by higher values of Triangular than *S. anguivi* and was less ellipsoid (i.e., higher Ellipsoid values) than the rest of groups, groups Aculeatum and Kumba were less circular (i.e., higher Circular values), had higher Shouder Height, Eccentricity, Fruit Shape Index Internal and Eccentricity Area Index than the rest of groups, and *S. anguivi* was more Obovoid than groups Kumba and Shum (Table 5).

**Table 5:** **Mean values for scarlet eggplant complex groups (*S. aethiopicum* groups Aculeatum, Gilo, Kumba, and Shum, Intermediate between *S. aethiopicum* and *S. anguivi*, and *S. anguivi*) for the fruit descriptors for which significant (*P* < 0.05) differences have been found among group means**.

**Descriptors^a^**	***S. aethiopicum***				**Intermediate**	***S. anguivi***	**Prob. F**
	**Aculeatum**	**Gilo**	**Kumba**	**Shum**			
*n*	5	37	10	2	8	1	
Weight (g)^b^	28.4^c^	32.4^c^	166.6^d^	3.9^b^	4.8^b^	1.0^a^	<0.0001
Length (cm)^b^	2.62^cd^	4.03^d^	4.21^d^	1.43^ab^	2.20^bc^	1.10^a^	<0.0001
Breadth (cm)^b^	4.63^c^	4.09^c^	8.22^d^	2.02^b^	2.06^b^	1.16^a^	<0.0001
Perimeter (cm)^b^	13.5^c^	13.8^c^	22.5^d^	5.9^ab^	6.9^b^	4.0^a^	<0.0001
Area (cm^2^)^b^	9.7^c^	13.1^c^	25.2^c^	2.5^ab^	3.3^b^	1.2^a^	<0.0001
Width Mid-height (cm)^b^	4.59^b^	3.96^b^	7.41^c^	1.97^a^	2.00^a^	1.24^a^	<0.0001
Maximum Width (cm)^b^	4.61^b^	3.98^b^	7.54^c^	1.98^a^	2.02^a^	1.25^a^	<0.0001
Height Mid-width (cm)^b^	1.86^ab^	3.82^c^	2.92^bc^	1.39^a^	2.03^ab^	1.15^a^	<0.0001
Maximum Height (cm)^b^	2.71^cd^	4.00^d^	4.32^d^	1.48^ab^	2.07^bc^	1.17^a^	<0.0001
Fruit Shape Index External I	0.59^a^	1.06^b^	0.57^a^	0.76^ab^	1.04^b^	0.93^ab^	<0.0001
Fruit Shape Index External II	0.41^a^	1.03^b^	0.40^a^	0.73^ab^	1.03^b^	0.92^ab^	<0.0001
Proximal Fruit Blockiness	0.73^b^	0.65^ab^	0.71^b^	0.69^ab^	0.62^ab^	0.57^a^	0.0235
Distal Fruit Blockiness	0.72^b^	0.65^ab^	0.57^a^	0.64^ab^	0.60^a^	0.64^ab^	0.0035
Fruit Shape Triangle	1.01^ab^	1.01^ab^	1.33^b^	1.07^ab^	1.10^ab^	0.90^a^	0.0001
Ellipsoid^b^	0.09^a^	0.04^a^	0.18^b^	0.04^a^	0.04^a^	0.02^a^	<0.0001
Circular^b^	0.29^b^	0.10^a^	0.25^b^	0.10^a^	0.08^a^	0.02^a^	<0.0001
Rectangular	0.57^b^	0.51^ab^	0.54^ab^	0.50^ab^	0.48^a^	0.50^ab^	0.0007
Shoulder Height^b^	0.11^b^	0.02^a^	0.08^b^	0.02^a^	0.01^a^	0.00^a^	<0.0001
Obovoid	0.05^ab^	0.08^ab^	0.03^a^	0.03^a^	0.07^ab^	0.13^b^	0.0073
Ovoid	0.08^ab^	0.06^ab^	0.16^b^	0.10^ab^	0.08^ab^	0.01^a^	<0.0001
Width Widest Pos	0.47^ab^	0.50^b^	0.43^a^	0.46^ab^	0.49^ab^	0.49^b^	<0.0001
Eccentricity	0.55^a^	0.75^b^	0.55^a^	0.76^b^	0.78^b^	0.78^b^	<0.0001
Fruit Shape Index Internal	0.41^a^	1.02^b^	0.41^a^	0.72^ab^	1.03^b^	0.92^b^	<0.0001
Eccentricity Area Index	0.56^b^	0.41^a^	0.54^b^	0.42^a^	0.39^a^	0.39^a^	<0.0001

a *Means within rows separated by different letters are significantly different at P < 0.05, according to the Student-Newman-Keuls test*.

b *In order to avoid scaling effects caused by accession means being proportional to standard deviations, ANOVAs were performed on log transformed data*.

The first and second components of the PCA accounted, respectively, for 33.7 and 16.6% of the total variation among accession means (Table 6). The first component was positively correlated to elongated fruits (Fruit Shape Index External I and II, and Fruit Shape Index Internal), Width Widest Pos, and Eccentricity and negatively to number of flower parts (sepals, petals, stamens), fruit size (except for the fruit length traits), and fruits less ellipsoid and circular (i.e., higher Ellipsoid and Circular values), and with high values for Shoulder Height, Ovoid, and Eccentricity Area Index (Table 6). The second component was positively correlated with anthocyanin intensity traits, prickliness traits, Number of Flowers per Inflorescence, and Distal Fruit Blockiness and negatively with Angle between Main Branches and with traits related to elongated (Length, Height Mid-width, Maximum Height, Fruit Shape Index External I and II, and Fruit Shape Internal) and large fruits (Perimeter and Area) (Table 6).

**Table 6:** **Correlation coefficients between plant and fruit descriptors and the two first principal components the for scarlet eggplant complex (*S. aethiopicum* and *S. anguivi*)**.

**Descriptor**	**First principal component**	**Second principal component**
**PLANT DESCRIPTORS**		
Hypocotyl Anthocyanins Intensity		0.198
Shoot Tip Anthocyanins Intensity		0.218
Angle Between Main Branches		−0.160
Leaf Prickles		0.230
Length of Largest Leaf Prickle		0.230
Number of Flowers per Inflorescence		0.233
Number of Sepals	−0.193	
Number of Petals	−0.206	
Number of Stamens	−0.203	
**FRUIT DESCRIPTORS**		
Weight	−0.194	
Length		−0.312
Breadth	−0.231	
Perimeter	−0.205	−0.191
Area	−0.186	−0.218
Width Mid-height	−0.232	
Maximum Width	−0.233	
Height Mid-width		−0.315
Maximum Height		−0.309
Fruit Shape Index External I	0.182	−0.185
Fruit Shape Index External II	0.197	−0.177
Proximal Fruit Blockiness	−0.160	
Distal Fruit Blockiness		0.167
Ellipsoid	−0.197	
Circular	−0.200	
Shoulder Height	−0.195	
Ovoid	−0.172	
Width Widest Pos	0.169	
Eccentricity	0.233	
Fruit Shape Index Internal	0.202	−0.178
Eccentricity Area Index	−0.231	
Variance Explained (%)	33.7	16.6

*Only those correlations with absolute values ≥0.15 have been listed*.

**Figure 4:**
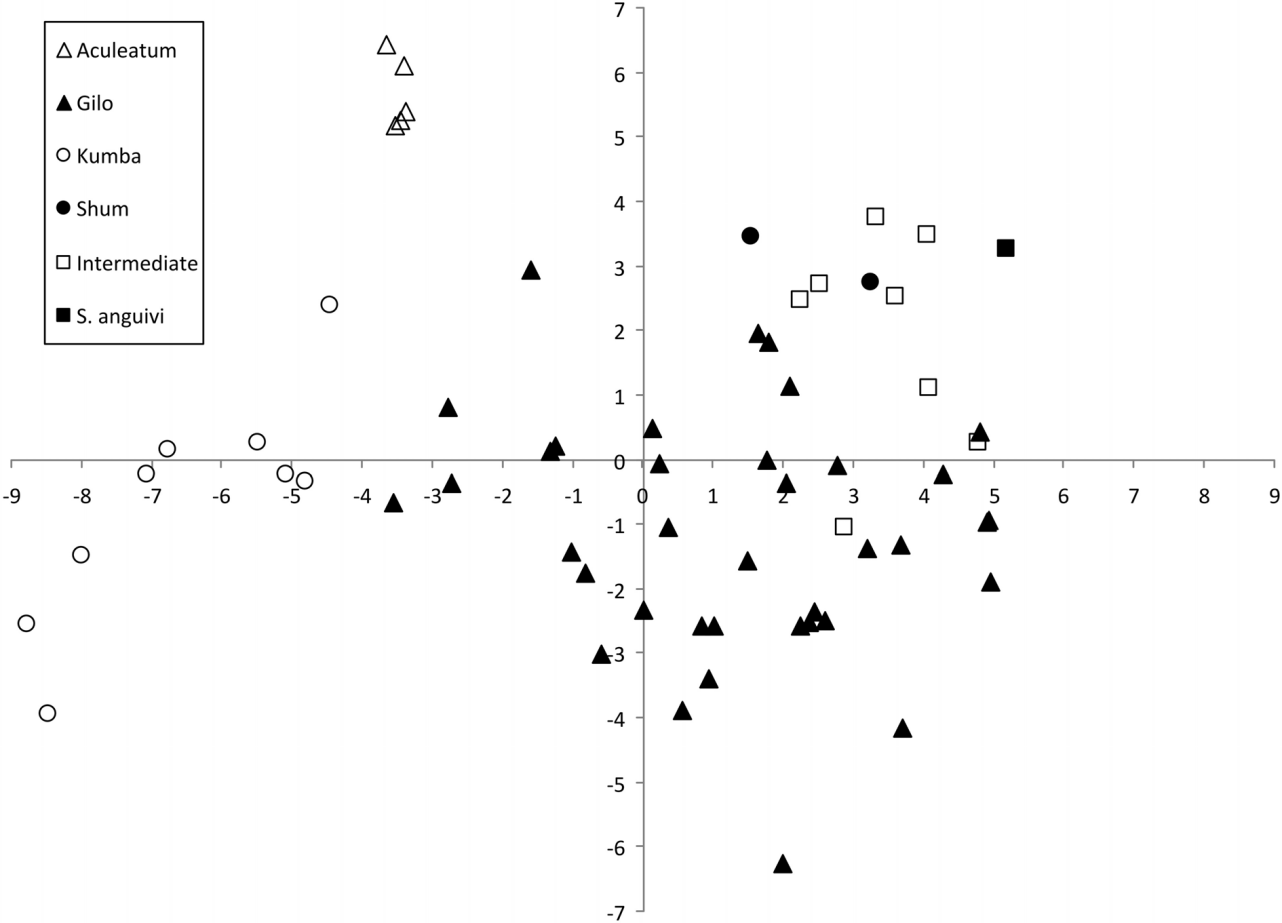
**Similarities based on 18 plant and 27 fruit descriptors among 63 scarlet eggplant complex (*S. aethiopicum* and *S. anguivi*) accessions represented on the two first principal components of PCA**. First and second components account for 33.7 and 16.6% of the total variation, respectively. The six groups considered are represented by different symbols: *S. aethiopicum* groups Aculeatum (open triangle), Gilo (filled triangle), Kumba (open circle), and Shum (filled circle); Intermediate between *S. aethiopicum* and *S. anguivi* (open square); and, *S. anguivi* (filled square).

The projection of the accessions on a two-dimensional PCA plot showed that accessions of the different scarlet eggplant complex groups plotted in different areas of the graph, although the Intermediate group overlapped with several of the other groups (Figure 4). The Aculeatum group had a low dispersion and all accessions presented negative values for the first component and highly positive values for the second component. The Gilo group presented the largest dispersion; however, despite this wide dispersion it overlapped only with some accessions of the Intermediate group. Kumba group accessions presented intermediate values for the first component and high negative values for the second component and display an intermediate level of dispersion compared to Aculateum and Gilo groups in the PCA graph. The small fruited Shum, Intermediate and *S. anguivi* groups presented a combination of high values for the first component (in particular *S. anguivi*) with moderate, generally positive, values for the second component. The Shum group and *S. anguivi* were separated from each other and from the Gilo group, but the Intermediate group overlapped with part of the areas where the Shum group accessions and small fruited accessions of the Gilo group plot and is also situated in the area intermediate between *S. anguivi*, Shum, and Gilo groups (Figure 4).

### Differences and relationships among gboma eggplant groups

Significant (*P* < 0.05) differences between *S. macrocarpon* and *S. dasyphyllum* were found only for three morphological traits (Table 7). In this respect, *S. macrocarpon* presented significantly less bullate leaves (Leaf Surface Shape) and lower prickliness (Leaf Prickless and Length of Largest Leaf Prickle) than *S. dasyphyllum*. Regarding fruit traits, significant differences between the two gboma eggplant groups were found for eleven traits. *Solanum macrocarpon* fruits presented significantly larger fruits (higher values for seven out of the eight fruit size traits, the exception being Height Mid-width), more ovoid (lower values for Obovoid and higher for Ovoid), and with lowest values for the ratio of the height at which the maximum width occurs (Width Widest Pos) than those of *S. dasyphyllum* (Table 7).

**Table 7:** **Mean values for gboma eggplant complex groups (*S. macrocarpon* and *S. dasyphyllum*) for the plant and fruit descriptors for which significant (*P* < 0.05) differences have been found among group means**.

**Descriptors^a^**	**Gboma eggplant**		**Prob. F**
	***S. macrocarpon***	***S. dasyphyllum***	
*n*	11	1	
Leaf Surface Shape	4.64^a^	9.00^b^	0.0061
Leaf Prickles	0.99^a^	9.00^b^	0.0004
Length of Largest Leaf Prickle (cm)	0.33^a^	1.45^b^	0.0442
Weight (g)^b^	119.1^b^	21.9^a^	0.0026
Length (cm)^b^	5.18^b^	2.97^a^	0.0068
Breadth (cm)^b^	6.92^b^	3.83^a^	0.0075
Perimeter (cm)^b^	21.0^b^	11.9^a^	0.0136
Area (cm^2^)^b^	28.8^b^	9.3^a^	0.0140
Width Mid-height (cm)^b^	6.80^b^	3.79^a^	0.0206
Maximum Width (cm)^b^	6.85^b^	3.81^a^	0.0199
Maximum Width (cm)^b^	5.14^b^	3.03^a^	0.0371
Obovoid	0.01^a^	0.05^b^	0.0093
Ovoid	0.16^b^	0.08^a^	0.0064
Width Widest Pos	0.44^a^	0.49^b^	0.0488

a *Means within rows separated by different letters are significantly different at P < 0.05, according to the Student-Newman-Keuls test*.

b *In order to avoid scaling effects caused by accession means being proportional to standard deviations, ANOVAs were performed on log transformed data*.

The first and second components of the PCA accounted, respectively, for 31.3 and 22.5% of the total variation among accession means (Table 8). The first component was positively correlated to prickliness (Leaf Prickles), elongated fruits (Fruit Shape Index External II), Width Widest Pos, and Eccentricity, and negatively to number of flower parts (Number of Petals, Number of Stamens), Corolla Diameter, leaf blade size, fruit size (except for the fruit length traits), and fruits more triangular, less ellipsoid and circular (i.e., higher Ellipsoid and Circular values), and with high values for Ovoid, and Eccentricity Area Index (Table 8). The second component was positively correlated with Plant Height, Stem Diameter, Number of Flowers per Inflorescence, Distal Fruit Blockiness and Proximal Eccentricity and negatively with anthocyanins intensity, traits related to elongated (Length, Height Mid-width, Maximum Height, Fruit Shape Index External I and II) and large fruits (Perimeter and Area) (Table 8).

**Table 8:** **Correlation coefficients between plant and fruit descriptors and the two first principal components for the gboma eggplant complex (*S. macrocarpon* and *S. dasyphyllum*)**.

**Descriptor**	**First principal component**	**Second principal component**
**PLANT DESCRIPTORS**		
Plant Height		0.219
Hypocotyl Anthocyanins Intensity		−0.266
Shoot Tip Anthocyanins Intensity		−0.247
Stem Diameter		0.206
Leaf Blade Length	−0.212	
Leaf Blade Breadth	−0.156	
Leaf Prickles	0.150	
Number of Flowers per Inflorescence		0.207
Number of Petals	−0.172	
Number of Stamens	−0.161	
Corolla Diameter	−0.217	
**FRUIT DESCRIPTORS**		
Weight	−0.216	
Length		−0.265
Breadth	−0.243	
Perimeter	−0.212	−0.154
Area	−0.201	−0.154
Width Mid-height	−0.238	
Maximum Width	−0.239	
Height Mid-width		−0.288
Maximum Height		−0.271
Fruit Shape Index External I		−0.255
Fruit Shape Index External II	0.152	−0.245
Proximal Fruit Blockiness		
Distal Fruit Blockiness		0.245
Fruit Shape Triangle	−0.154	
Ellipsoid	−0.169	
Circular	−0.168	
Ovoid	−0.197	
Width Widest Pos	0.224	
Eccentricity	0.230	
Proximal Eccentricity		0.194
Fruit Shape Index Internal	0.152	
Eccentricity Area Index	−0.220	
Variance Explained (%)	31.3	22.5

*Only those correlations with absolute values ≥0.15 have been listed*.

**Figure 5:**
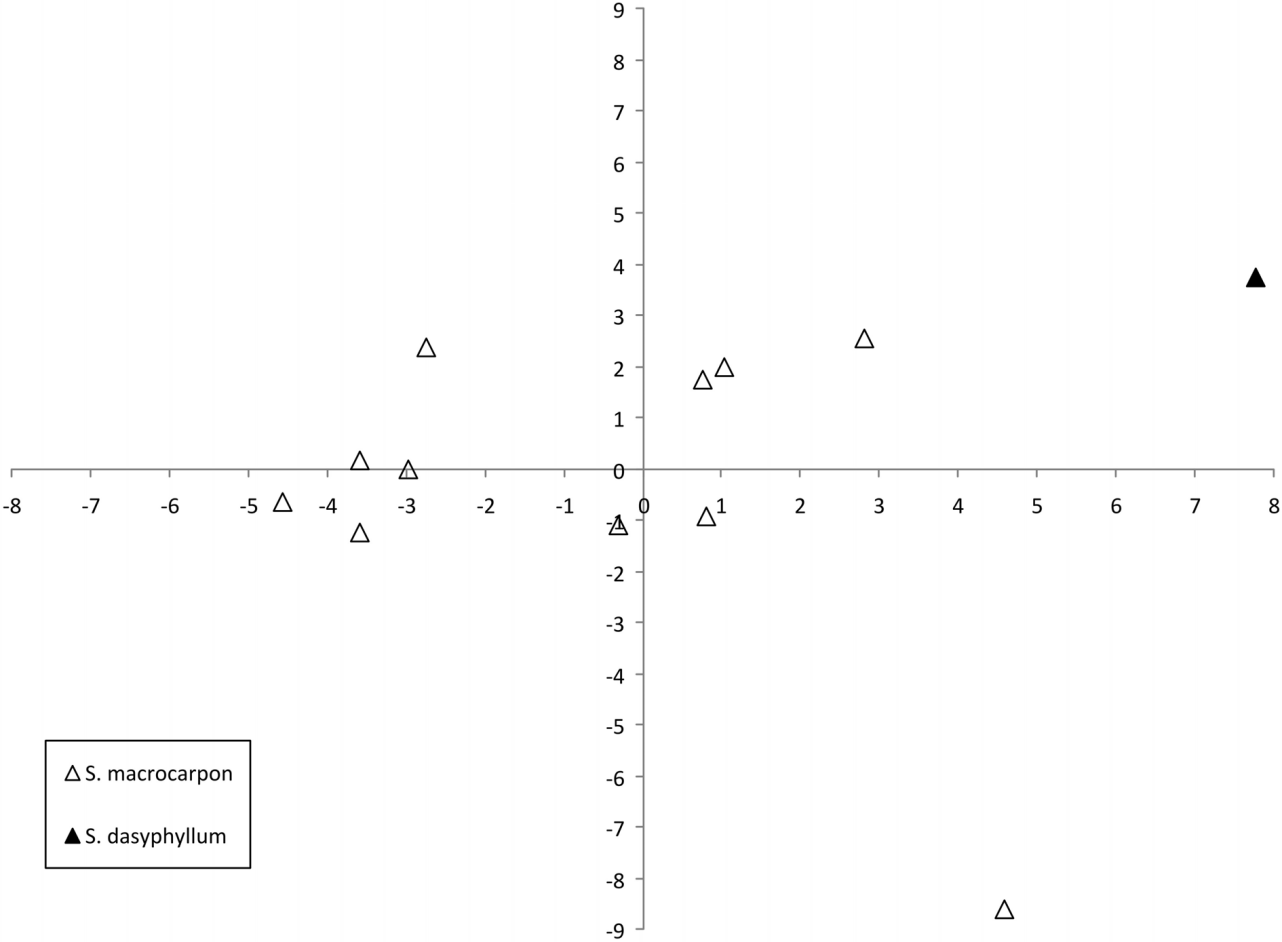
**Similarities based on 18 plant and 27 fruit descriptors among 12 gboma eggplant complex (*S. aethiopicum* and *S. anguivi*) accessions represented on the two first principal components of PCA**. First and second components account for 31.3 and 22.5% of the total variation, respectively. The two species are represented by different symbols: *S. macrocarpon* (open triangle), and *S. dasyphyllum* (filled triangle).

The projection of the accessions on a two-dimensional PCA plot clearly shows that accessions of *S. macrocarpon* and *S. dasyphyllum* groups plot in different areas of the graph (Figure 5). The single accession of *S. dasyphyllum* presents the highest values for the first and second components. With the exception of one odd accession, all the *S. macrocarpon* accessions present intermediate values for the first component. The odd *S. macrocarpon* accession, with an extremely low value for the second component is distinct from the others in having elongated fruit shape.

## Discussion

Scarlet and gboma eggplants are important vegetables in tropical sub-Saharan Africa but have received little attention from the formal breeding sector (Lester and Thitai, 1989; Schippers, 2000; Seck, 2000; Adeniji and Aloyce, 2012; Prohens et al., 2012). This has allowed the on-site conservation of a large number of local varieties which, together with accessions conserved in germplasm banks, represent genetic resources for the enhancement of both crops (Lester et al., 1990; Bukenya and Carasco, 1994; Schippers, 2000; Sekara et al., 2007). The detailed morphological characterization of germplasm collections will allow studying the diversity and identification of potentially interesting accessions for selection and breeding, as well as devising strategies for conservation and management of germplasm (Furbank and Tester, 2011). Also, given that both crops and their wild relatives form part of the secondary genepool of common eggplant, information on the phenotypic diversity of scarlet and gboma eggplants may be of interest for common eggplant breeding (Daunay et al., 1991; Oyelana and Ugborogho, 2008; Prohens et al., 2012; Khan et al., 2013).

Morphological characterization is essential for the identification of valuable germplasm accessions as well as for typification and classification of accessions in cultivar groups (Spooner et al., 2003). Characterization of cultivated eggplants and wild relatives has usually been performed with conventional morphological descriptors highly heritable and simple to evaluate (IBPGR, 1990; Prohens et al., 2005; van der Weerden and Barendse, 2007; Polignano et al., 2010; Prohens et al., 2012). These descriptors are very useful but have some limitations especially for fruit shape characterization, which is one of the most important traits in a variety of any of the cultivated eggplant species and for which great diversity exists (Adeniji and Aloyce, 2012; Hurtado et al., 2013). Here we have complemented a standard morphological characterization with a fruit shape phenomics characterization using the high-throughput phenomics Tomato Analyzer (Brewer et al., 2006, 2007; Gonzalo and van der Knaap, 2008; Rodríguez et al., 2010), which has allowed the automated acquisition of multiple data of different fruit shape characteristics in both scarlet and gboma eggplants complexes. Combination of both types of data has allowed identification of multiple traits which distinguish clearly not only both crops and the cultivated species from the wild relatives, but also cultivar groups, which is not always possible using conventional descriptors (Polignano et al., 2010; Adeniji et al., 2012, 2013), as well as to describe the diversity present for traits of interest for selecting and developing improved materials in both crops. Descriptors presenting highly significant differences among groups and which plot in different parts of the PCA graph (i.e., descriptors that do not present high correlation values) would be the most informative for distinguishing between cultivar groups.

Scarlet and gboma eggplants are classified in different botanical sections within *Solanum* subgenus *Leptostemonun* (Lester, 1986; Lester and Daunay, 2003; Lester et al., 2011; Edmonds, 2012). Our results confirm that the scarlet and gboma eggplant complexes differ in many morphological differences, both for plant and fruit traits of agronomic interest. Polignano et al. (2010) also found that the cultivated *S. aethiopicum* and *S. macrocarpon* presented considerable differences for agronomic descriptors. Although some differences considered as significant (*P* < 0.05) might have resulted from false positives derived from multiple testing (Snedecor and Cochran, 1989), most of them have been highly significant (*P* < 0.001), indicating that even with highly stringent tests they would have been significant, revealing that very likely they correspond to real differences. This differentiation is also confirmed at the molecular and chemical composition levels (Furini and Wunder, 2004; Polignano et al., 2010; Sánchez-Mata et al., 2010; Vorontsova et al., 2013). Given that both crops can be intercrossed and hybrids have intermediate fertility (Daunay et al., 1991; Oyelana and Ugborogho, 2008), scarlet and gboma eggplants could be used for reciprocal breeding in order to introgress traits of interest from one species into the other (Prohens et al., 2012).

We have found a large diversity in both scarlet and gboma eggplants complexes for plant and fruit traits, with wide ranges of variations for most descriptors, confirming that they are hypervariable (Lester and Niakan, 1986; Lester et al., 1986; Bukenya and Carasco, 1994; Schippers, 2000). The variation of *Solanum aethiopicum* is so high that the different cultivar groups have, in the past, been considered as different species (Lester, 1986; Lester et al., 1986, 2011). Our combined study of conventional and Tomato Analyzer descriptors, together with multivariate PCA results, shows that each of the *S. aethiopicum* cultivar groups as well as *S. anguivi* are distinguished by many traits, which supports Lester (1986) view that each of the *S. aethiopicum* cultivar groups and the wild ancestor *S. anguivi* are characterized by a specific syndrome of characteristics. As expected, the wild *S. anguivi*, the intermediate forms between *S. aethiopicum* and *S. anguivi*, as well as the *S. aethiopicum* Shum group, which is only used for the leaves, have small fruits (Lester and Niakan, 1986; Lester et al., 1986, 1990; Schippers, 2000). Also, the gboma eggplant complex has proved to be highly diverse (Bukenya and Carasco, 1994; Lester et al., 1990; Polignano et al., 2010). Apart from leaf surface shape and prickliness, the differences observed between *S. macrocarpon* and *S. dasyphyllum* correspond to fruit traits evaluated with Tomato Analyzer. Most of the traits for which differences have been found among scarlet eggplant complex groups (including *S. anguivi*) as well as between *S. macrocarpon* and *S. dasyphyllum* correspond to fruit shape traits identified using the Tomato Analyzer tool, showing the potential of this phenomics tool for fruit shape characterization in eggplants (Prohens et al., 2012; Hurtado et al., 2013). In fact, while no significant differences were found for conventional plant descriptors between *S. aethiopicum* Gilo and Shum groups on one hand, as well as between the Intermediate group and *S. anguivi* on the other, the Tomato Analyzer characterization of fruit shape has allowed the detection of significant differences for fruit shape traits among them.

Apart from the differences among *S. aethiopicum* groups, a wide diversity has been found within each of them, as well as within *S. macrocarpon*. Within *S. aethiopicum*, the largest diversity has been found in the Gilo group, which is in agreement with previous observations and also with the fact that it is the most spread and important cultivar group (Lester et al., 1986, 1990; Schippers, 2000; Polignano et al., 2010; Sunseri et al., 2010). The Kumba group has been found to be less diverse that the Gilo group. The fact that the characteristic highly furrowed and flattened fruits of the Kumba group may be the result of a mutation similar to that of the tomato *FASCIATED* mutation, which is responsible for a high degree of fasciation in this crop (Monforte et al., 2014), may account for the lower degree of diversity compared to the Gilo group (Lester et al., 1986). Amazingly, a low diversity has been found within the Aculeatum group. This group is not commonly found in Africa and it has been hypothesized that it was created in Europe for ornamental purposes after crossing *S. anguivi* and *S. aethiopicum* group Kumba (Lester et al., 1986; Schippers, 2000), which would explain its low diversity.

Lester et al. (1986, 1990) reported that some accessions were intermediate in characteristics between *S. anguivi* and *S. aethiopicum*. In our case, we have found several of them, which presented some key traits used for classification that were typical of *S. aethiopicum* while others were characteristic of *S. anguivi*. These materials plotted between *S. anguivi* and *S. aethiopicum*, having some overlap with the latter. Intermediate forms may represent primitive or semi-domesticated weedy forms that are formed by occasional hybridization, as the area of natural distribution of the wild ancestor presents a high degree of overlapping with the area of distribution of the crop (Lester and Niakan, 1986; Lester et al., 1986, 1990). This intermediate forms very likely favor the flux of genes from the wild *S. anguivi* into the cultivated *S. aethiopicum*, contributing to a high genetic background and diversity.

*Solanum macrocarpon* accessions have also been very variable for the traits studied. An important diversity may be caused by the fact that in this crop some accessions are used for the leaves, others for the fruits, and others for both plant organs (Lester et al., 1990; Schippers, 2000; Maundu et al., 2009). Therefore, it is expected that accessions used for the leaves will have smaller fruits than those used for the fruits. Also, although a characteristic typical of *S. macrocarpon* is having fruits flattened or subspherical, an accession with elongated fruits has been found. It remains to be investigated if the elongated fruit of this odd accession is caused by a mutation similar to the *SUN* mutation of tomato, which results in extremely elongated fruits (Monforte et al., 2014).

The phenotypic results obtained have important implications for germplasm conservation and breeding (Furbank and Tester, 2011). The high diversity found indicates that a large number of accessions will need to be conserved in germplasm banks or represented in core collections in order to have a good representation of the phenotypic variation found in both species (Odong et al., 2013). The characterization data and the multivariate analysis performed may be useful to select a subset of accessions that represent most of the morphological diversity of both complexes. At the selection and breeding level, considerable phenotypic differences among and within groups may be used for selection of the best accessions or to select parents for obtaining F1 hybrids heterotic for yield or with intermediate or new characteristics (Lester and Thitai, 1989; Seck, 2000; Adeniji and Aloyce, 2012).

In conclusion, we have found that the combined utilization of conventional and Tomato Analyzer phenomics descriptors is a powerful tool for studying the diversity and relationships of scarlet and gboma eggplants complexes. In particular, Tomato Analyzer allows the detailed description of fruit characteristics and the differentiation of cultivar groups in which few plant morphological differences are found. The detailed characterization information on the germplasm collections will be useful for the enhancement of both crops, including the conservation of genetic resources, selection and breeding.

### Conflict of interest statement

The authors declare that the research was conducted in the absence of any commercial or financial relationships that could be construed as a potential conflict of interest.
